# Recurrent macular hole retinal detachment in a patient with pathologic myopia treated by additional intravitreal silicone oil injection: A case report

**DOI:** 10.1097/MD.0000000000025465

**Published:** 2021-04-09

**Authors:** Do Yeon Kim, Yu Cheol Kim

**Affiliations:** Department of Ophthalmology, Keimyung University School of Medicine, Daegu, Republic of Korea.

**Keywords:** macular hole retinal detachment, pathologic myopia, silicone oil, staphyloma, tamponade

## Abstract

**Rationale:**

Treatment of macular hole retinal detachment (MHRD) in patients with pathologic myopia may require multiple surgeries due to the risk of surgical failures or recurrences. Intravitreal silicone oil injection before an additional surgery may be another option for recurrent MHRD in aphakic eyes, but this procedure is rarely performed.

**Patient concerns:**

A 69-year-old man visited the hospital with a chief complaint of metamorphopsia in his right eye for 5 days. The right eye had undergone a cataract extraction 5 years prior and an Nd:YAG laser capsulotomy 1 year prior. The axial length was 36.18 mm; the fundus examination and optical coherence tomography (OCT) revealed inferior retinal detachment with a macular hole involving the posterior pole. Pars plana vitrectomy (PPV) with internal limiting membrane (ILM) peeling, endolaser photocoagulation, and silicone oil tamponade were performed. Five months after the surgery, the retina was detached, and a macular hole was observed.

**Diagnosis:**

Recurrent MHRD in a patient with pathologic myopia.

**Intervention:**

PPV with ILM peeling, endolaser photocoagulation, and silicone oil tamponade at the initial visit and additional intravitreal silicone oil injection (0.5 ml) at follow-up visits.

**Outcomes:**

The retina was well-attached until 5 months after the additional intravitreal silicone oil injection.

**Lessons:**

Additional intravitreal silicone oil injection can be a good option for treating MHRD in aphakic eyes if the detachment of the retina is dependent on posturing. The surgeon should consider the volume of silicone oil or postoperative posturing in the treatment of MHRD.

## 1 Introduction

Myopia is becoming increasingly prevalent worldwide and is an epidemic in East Asian countries.^[1]^ Among young adults in East Asian countries, the prevalence of myopia and high myopia is 83% to 97% and 7% to 22%, respectively.^[2–4]^ Adults with high myopia and excessive axial length are at high risk of developing pathologic myopia, one of the leading causes of irreversible vision loss.^[5]^ Pathologic myopia is defined as high myopia-associated structural changes in the posterior segment of the eye, leading to permanent vision impairment. Posterior staphyloma is a hallmark of pathologic myopia, defined as an outpouching of the ocular wall with a curvature radius less than the surrounding regions.^[6]^ Posterior staphyloma and excessive globe elongation may lead to complications. Macular hole retinal detachment (MHRD) is one of the most vision-threatening complications of pathologic myopia. The important causative factors of MHRD might be related to the tangential and anteroposterior traction due to the presence of a posterior staphyloma combined with weakened retinal adhesion caused by retinal pigment epithelium atrophy.^[7]^ These background pathologies make MHRD a surgical challenge in patients with high myopia.

Various surgical techniques to treat MHRD have been reported, such as episcleral macular buckling. Gomvers and Machemer^[8]^ reported pars plana vitrectomy (PPV) with gas tamponade for the first time and found it to be a useful technique. Several additional techniques with PPV have been reported, such as silicone oil tamponade, laser photocoagulation around the macular hole (MH), epiretinal membrane peeling, and internal limiting membrane (ILM) peeling, and inverted ILM flap.^[9]^ However, despite these procedures, re-detachment may still develop, and some eyes require multiple surgeries to achieve reattachment.

Herein, we report a case of recurrent MHRD in pathologic myopia treated successfully only with an additional intravitreal silicone oil injection.

## 2 Case summary

**Figure 1:**
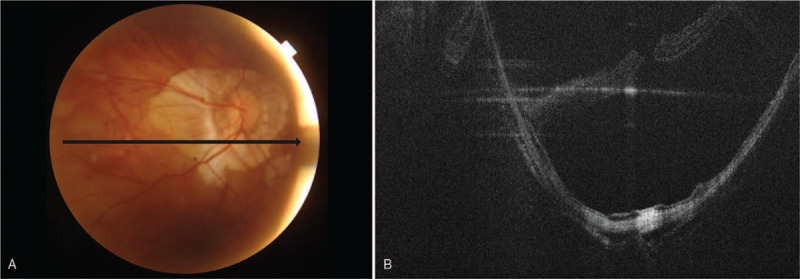
Initial findings of the right eye. (A) Fundus photography reveals myopic degeneration with peripapillary atrophy. (B) Optical coherence tomography shows retinal detachment involving posterior pole with staphyloma.

A 69-year-old man visited the hospital for a complaint of metamorphopsia (oculus dexter [OD]) for 5 days. The right eye underwent cataract lens extraction 5 years before and neodymium-doped yttrium aluminum garnet (Nd:YAG) capsulotomy 1 year before this visit. On admission, the axial length using Zeiss IOL Master 700 (Carl Zeiss Meditec AG, Jena, Germany) was 36.18 mm (OD) and 34.43 mm (oculus sinister [OS]); the best-corrected visual acuity (BCVA) by Snellen chart was 0.7 (OD) and finger count 30 cm (OS); the intraocular pressure was 13 mm Hg (OD) and 12 mm Hg (OS); the fundus examination and optical coherence tomography (OCT) revealed inferior retinal detachment with MH involving posterior pole (OD) (Fig. 1) and myopic degeneration with staphyloma (OS). At that visit, we performed a 25-gauge pars plan vitrectomy (PPV) with an ILM peeling using indocyanine green, endolaser photocoagulation, and 5700-centistoke silicone oil (Oxane, Bausch & Lomb, New York, NY) tamponade was performed. Postoperatively, the retina was well-attached, but the MH was not sealed (Fig. 2 A). However, the patient complained that the vision (OD) was decreased in the morning and the issues were relieved in the evening 4 months after the surgery, although the retina was well-attached in OCT.

**Figure 2:**
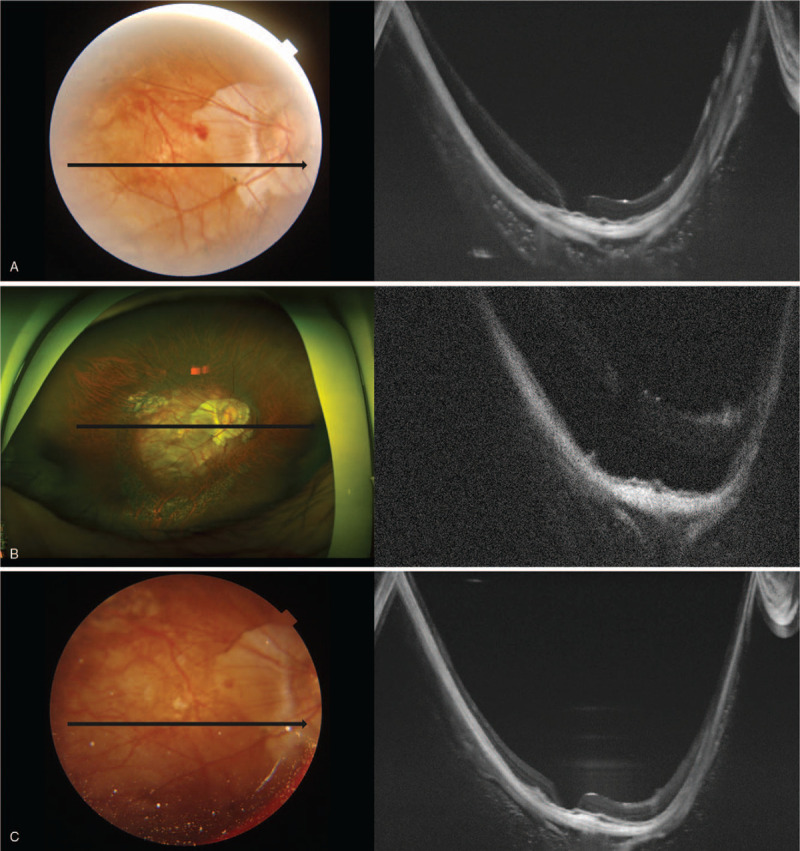
Series of optical coherence tomography examinations of the right eye after surgery. (A) the retina was well-attached, but the macular hole was not sealed. (B) 5 months after surgery, the retina was detached from the macular hole. (C) on the next day, the retinal detachment was relieved. (D) 4 days later, the retinal detachment was aggravated. (E) after 1 hour in the face-down position, the retinal detachment was relieved. (F) after the additional intravitreal silicone oil injection, the retina was well-attached for 6 months.

**Figure 2 (Continued):**
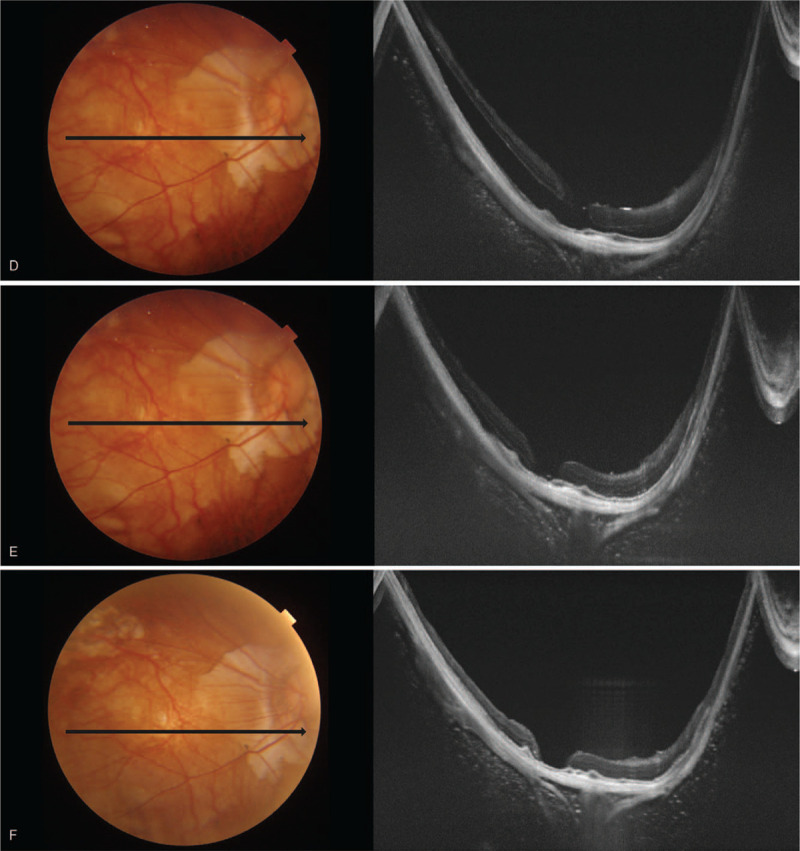
Series of optical coherence tomography examinations of the right eye after surgery. (A) the retina was well-attached, but the macular hole was not sealed. (B) 5 months after surgery, the retina was detached from the macular hole. (C) on the next day, the retinal detachment was relieved. (D) 4 days later, the retinal detachment was aggravated. (E) after 1 hour in the face-down position, the retinal detachment was relieved. (F) after the additional intravitreal silicone oil injection, the retina was well-attached for 6 months.

The patient complained of sudden decreased visual acuity (OD) 5 mo after surgery; the BCVA (OD) was hand motion; the retina was detached with an MH (Fig. 2 B), with an intraocular pressure of 10 mm Hg. On the next day, the retinal detachment was relieved (Fig. 2 C), and the patient was advised to maintain a face-down position. The retinal detachment had aggravated 4 days later (Fig. 2 D), but it was relieved after lying in a face-down position for 1 hour (Fig. 2 E). The authors injected 0.5 ml of silicone oil into the vitreous cavity through the anterior chamber. The retina was well-attached until the last follow-up at 6 months after the additional intravitreal silicone oil injection (Fig. 2 F), but the BCVA was 0.06 at the last visit.

## 3 Discussion

In highly myopic eyes, the surgical procedure for MHRD remains particularly challenging because of the presence of chorioretinal atrophy, poor retinal adhesion to the underlying pigment epithelium, and posterior staphyloma that produces inverse traction to overcome retinal adhesion. Silicone oil tamponade was used in previous studies, and the initial retinal reattachment rate was 62% to 92%.^[10–14]^

Buoyancy arises from the difference in specific gravity between the vitreous humor and silicone oil. Compared with water (specific gravity, 1.00), the specific gravity of vitreous humor is slightly higher, while that of silicone oil is a little lower (0.97). Therefore, silicone oil floats inside the vitreous cavity, and the upward force is defined as buoyancy. This force is highest at the apex and gradually decreases to zero at the horizontal meniscus. Accordingly, the face-down position after MHRD surgery may maximize the tamponade effect of silicone oil.

Although blocking the communication of the vitreous humor through the MH and pushing the retina onto the eyeball wall by gas or silicone oil is essential for the treatment of MHRD, the essential process is not easy to achieve in pathologic myopia because it has staphyloma as well as a large vitreous cavity. The MH is usually located on the staphyloma. Nagra et al^[15]^ suggested that total ocular volume increases as axial length or corneal radius increases. The axial length in the current case was 36.18 mm, suggesting a much larger ocular volume than that for a normal eyeball. Nakanishi et al^[16]^ suggested that staphyloma's depth is associated with retinal adhesion rather than the extent. This finding is presumably because the larger the difference in the radius between the eyeball and staphyloma, the less the tamponade effect on the staphyloma.

**Figure 3:**
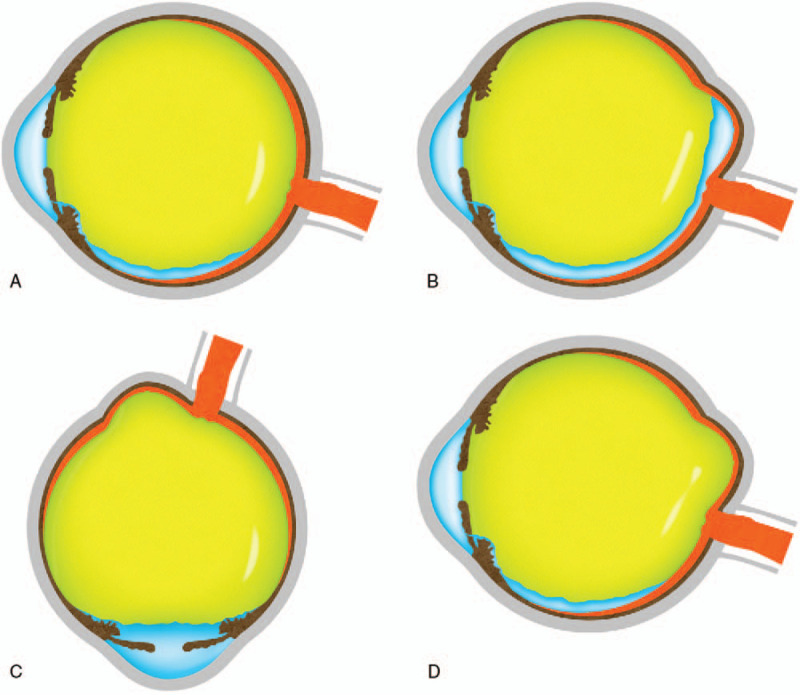
Mimetic images showing various tamponade states depend on the posture, lens state, and silicone oil volume. (A) normal-sized eye with silicone oil tamponade at the sitting-up position, (B) pathologic myopic eye with insufficient silicone oil at sitting-up position, (C) pathologic myopic eye with silicone oil at face-down position, (D) pathologic myopia with sufficient silicone oil at the sitting-up position.

For these reasons, pathologic myopic eyes have more difficulties with tamponade than do normal-sized eyes (Fig. 3A, 3B). This observation means that the tamponade effect in pathologic myopia may be more dependent on the postoperative position or the volume of silicone oil and more vulnerable to non-restrictive posturing or insufficient volume of silicone oil (Fig. 3B). This phenomenon and tendency are assumed to be more evident in aphakic eyes in which the tamponade material may not be restricted to the posterior chamber. Although Nishimura el^[13]^ reported that PPV with ILM peeling and silicone oil tamponade in MHRD of highly myopic eyes achieved a high retinal reattachment rate without any postoperative posturing restriction, and they had no aphakic case. The current case might have an insufficient volume of silicone oil to maintain the retinal attachment without position restriction. However, the retinal attachment was achieved when the patient assumed a face-down position immediately after the surgery (Fig. 3C). The attachment force might not have been strong enough to maintain retinal adhesion without tamponade due to retinal pigment epithelial atrophy and inverse traction of the staphyloma. During sleep, the face-up position might have induced shallow retinal detachment, and the patient thus felt that the vision worsened in the morning. During the daytime, the sitting-up position or face-down position might have induced the retinal attachment, and the patient felt the vision was getting better in the evening. The daily repetition of the retinal detachment and attachment might have increased the subretinal fluid, which made complete reattachment during daytime by posturing impossible. However, the larger volume of silicone oil achieved by additional intravitreal silicone oil injection made it possible (Fig. 2 F and Fig. 3D).

Our case's limitation is that it had a relatively short-term follow-up period. However, to the best of our knowledge, this is the first case report of recurrent MHRD in pathologic myopia treated successfully with only an additional intravitreal silicone oil injection.

## 4 Conclusion

In conclusion, the surgeon should be careful about the volume of silicone oil or postoperative posturing in the treatment of MHRD. Although MHRD in pathologic myopia may require multiple surgeries after initial failures or recurrences, the current case shows that additional intravitreal silicone oil injection can be a good option for treating MHRD, especially in aphakic eyes, if the detachment of the retina is dependent on posturing.

## Author contributions

**Conceptualization:** Do Yeon Kim, Yu Cheol Kim.

**Data curation:** Do Yeon Kim, Yu Cheol Kim.

**Formal analysis:** Do Yeon Kim, Yu Cheol Kim.

**Funding acquisition:** Yu Cheol Kim.

**Investigation:** Do Yeon Kim, Yu Cheol Kim.

**Methodology:** Yu Cheol Kim.

**Project administration:** Yu Cheol Kim.

**Resources:** Yu Cheol Kim.

**Software:** Yu Cheol Kim.

**Supervision:** Yu Cheol Kim.

**Validation:** Yu Cheol Kim.

**Visualization:** Do Yeon Kim, Yu Cheol Kim.

**Writing – original draft:** Do Yeon Kim, Yu Cheol Kim.

**Writing – review & editing:** Yu Cheol Kim.

## References

[1] Morgan IG French AN Ashby RS. The epidemics of myopia: aetiology and prevention. Prog Retin Eye Res 2018;62:134–49. 28951126 10.1016/j.preteyeres.2017.09.004

[2] Jung S-K Lee JH Kakizaki H. Prevalence of myopia and its association with body stature and educational level in 19-year-old male conscripts in Seoul, South Korea. Invest Ophthalmol Vis Sci 2012;53:5579–83. 22836765 10.1167/iovs.12-10106

[3] Lee JH Jee D Kwon J-W. Prevalence and risk factors for myopia in a rural Korean population. Invest Ophthalmol Vis Sci 2013;54:5466–71. 23838769 10.1167/iovs.13-12478

[4] Lee Y-Y Lo C-T Sheu S-J. What factors are associated with myopia in young adults? A survey study in Taiwan Military Conscripts. Invest Ophthalmol Vis Sci 2013;54:1026–33. 23322575 10.1167/iovs.12-10480

[5] Wong Y-L Saw S-M. Epidemiology of pathologic myopia in Asia and worldwide. Asia Pac J Ophthalmol (Phila) 2016;5:394–402. 27898442 10.1097/APO.0000000000000234

[6] Ohno-Matsui K Jonas JB. Posterior staphyloma in pathologic myopia. Prog Retin Eye Res 2019;70:99–109. 30537538 10.1016/j.preteyeres.2018.12.001

[7] Kinoshita T Onoda Y Maeno T. Long-term surgical outcomes of the inverted internal limiting membrane flap technique in highly myopic macular hole retinal detachment. Arbeitsphysiologie 2017;255:1101–6. 10.1007/s00417-017-3614-0 28220252

[8] Gonvers M Machemer R. A new approach to treating retinal detachment with macular hole. Am J Ophthalmol 1982;94:468–72. 7137271 10.1016/0002-9394(82)90240-9

[9] Kakinoki M Araki T Iwasaki M. Surgical outcomes of vitrectomy for macular hole retinal detachment in highly myopic eyes: a multicenter study. Ophthalmol Retina 2019;3:874–8. 31257070 10.1016/j.oret.2019.04.026

[10] Hong N Huang B-S Tong J-P. Primary silicone oil tamponade and internal limiting membrane peeling for retinal detachment due to macular hole in highly myopic eyes with chorioretinal atrophy. BMC Ophthalmol 2015;15:165. 26560878 10.1186/s12886-015-0154-4 PMC4642637

[11] Mancino R Ciuffoletti E Martucci A. Anatomical and functional results of macular hole retinal detachment surgery in patients with high myopia and posterior staphyloma treated with perfluoropropane gas or silicone oil. Retina 2013;33:586–92. 23042102 10.1097/IAE.0b013e3182670fd7

[12] Nadal J Verdaguer P Canut MI. Treatment of retinal detachment secondary to macular hole in high myopia: vitrectomy with dissection of the inner limiting membrane to the edge of the staphyloma and long-term tamponade. Retina 2012;32:1525–30. 22466478 10.1097/IAE.0b013e3182411cb8

[13] Nishimura A Kimura M Saito Y. Efficacy of primary silicone oil tamponade for the treatment of retinal detachment caused by macular hole in high myopia. Am J Ophthalmol 2011;151:148–55. 21130979 10.1016/j.ajo.2010.07.023

[14] Xie A Lei J. Pars plana vitrectomy and silicone oil tamponade as a primary treatment for retinal detachment caused by macular holes in highly myopic eyes: a risk-factor analysis. Curr Eye Res 2013;38:108–13. 22992184 10.3109/02713683.2012.722742

[15] Nagra M Gilmartin B Logan NS. Estimation of ocular volume from axial length. Br J Ophthalmol 2014;98:1697–701. 24985726 10.1136/bjophthalmol-2013-304652

[16] Nakanishi H Kuriyama S Saito I. Prognostic factor analysis in pars plana vitrectomy for retinal detachment attributable to macular hole in high myopia: a multicenter study. Am J Ophthalmol 2008;146:198–204. 18547540 10.1016/j.ajo.2008.04.022

